# Molecular Characterization of E-Type Prostanoid Receptor 4 (EP4) from Ayu (*Plecoglossus altivelis*) and Its Functional Analysis in the Monocytes/Macrophages

**DOI:** 10.1371/journal.pone.0147884

**Published:** 2016-01-25

**Authors:** Ye-Jing Rong, Xin-Jiang Lu, Jiong Chen

**Affiliations:** 1 Laboratory of Biochemistry and Molecular Biology, School of Marine Sciences, Ningbo University, Ningbo, 315211, China; 2 Collaborative Innovation Center for Zhejiang Marine High-efficiency and Healthy Aquaculture, Ningbo University, Ningbo, 315211, China; Universidade Federal do Rio de Janeiro, BRAZIL

## Abstract

Prostaglandin E2 (PGE2) plays an important role in a broad spectrum of physiological and pathological processes by interacting with E-type prostanoid receptors (EPs). EP4 is one of four EP subtypes known to mediate the immune response in mammalian monocytes/macrophages. However, the precise function and characteristics of EP4 in fish remain unclear. In this study, we characterized a novel EP4-like (PaEP4L) gene from ayu, *Plecoglossus altivelis*. The cDNA sequence of PaEP4L is 2781 nucleotides (nts) in length, encoding a polypeptide of 459 amino acid residues with a calculated molecular weight of 51.17 kDa. Sequence comparison and phylogenetic tree analysis showed that PaEP4L shared 76% amino acid identity with that of the Atlantic salmon (*Salmo salar*). PaEP4L mRNA was detected by real-time quantitative PCR (QPCR) in all tested tissues and head kidney-derived monocytes/macrophages (MO/MФ). It varied greatly in liver, spleen and MO/MФ upon *Vibrio anguillarum* infection. Western blot analysis revealed a significant increase of PaEP4L in cell homogenates from ayu MO/MФ upon *V*. *anguillarum* infection. Moreover, anti-PaEP4L IgG reversed the down-regulation of interleukin 1β (IL-1β) and tumor necrosis factor α (TNF-α) mRNA expression as well as phagocytosis in ayu MO/MФ caused by PGE2. There were no significant differences in the respiratory burst response between PGE2 treated and untreated cells. We further found that cAMP mediated PGE2/PaEP4L signal in ayu MO/MФ. In conclusion, our results indicate that PaEP4L mediates PGE2 effects on ayu MO/MФ function, revealing that EP4 also plays a role in the modulation of cells of the fish’s innate immune system.

## Introduction

Fatty acids are not only a source of energy, but also signaling molecules and precursors for the synthesis of eicosanoids [1]. Studies have shown that fatty acids may affect a great number of immune parameters, such as lymphocyte proliferation, cytokine synthesis, natural killer (NK) cell activity, and phagocytosis [2]. Prostaglandin E2 (PGE2) is the most abundant eicosanoid in the inflammatory conditions stimulated by pathogen-associated molecular patterns (PAMPs) on cells of the innate immune system [3]. PGE2 can be produced by a variety of cells, such as macrophages, fibroblasts, and some types of malignant cells [4], and it can affect macrophage function in a variety of systems [5]. For example, PGE2 produced by bone marrow stromal cells is shown to promote IL-10 production from macrophages *in vivo* [6]. Furthermore, PGE2 leads to the inhibition of phagocytosis in pulmonary macrophages [7]. In addition, PGE2-dependent elevation of intracellular cyclic adenosine monophosphate (cAMP) in lipopolysaccharide (LPS)-stimulated macrophages resulted in a suppression of synthesis of pro-inflammatory cytokines, such as interleukin 1β (IL-1β) [8] and tumor necrosis factor α (TNF-α) [9, 10].

The biological actions of PGE2 are known to be mediated via one of four E-type prostanoid receptors (EPs), designated EP1, EP2, EP3 and EP4 in mammals [11]. PGE2 exerts anti-inflammatory effects by binding to EP4, thereby modulating macrophage functions. EP4 belongs to the seven-transmembrane G protein-coupled receptor (GPCR) superfamily. EP4 has an aspartate which is involved in receptor-ligand interaction [12] in the second transmembrane domain, and a pair of conserved cysteine residues which form a disulfide bond critical for stabilization of receptor conformation [12] in the second and third extracellular domains. The arginine in the seventh transmembrane domain may be the binding site of the prostanoids [13], which is conserved in all EP4 [14]. Activation of EP4 suppresses the release of cytokines and chemokines, such as TNF-α, IL-12, and MCP-1, from macrophages [14, 15]. In recent years, EP4 has been attracting considerable attention due to its immunomodulatory activity in the mammalian immune response, and its homologs have been identified in various fish and shellfish, such as zebrafish (*Danio rerio*) [16], Atlantic salmon (*Salmo salar*) [17], and Hong Kong oyster (*Crassostrea hongkongensis*) [18]. The tissue mRNA expression profile of Atlantic salmon EP4 has been studied [19, 20], and it is reported to be most abundantly expressed in the spleen followed by the head kidney, gill, and intestine, while the weakest expression is found in the heart and muscles [19, 20]. However, the role of fish EP4 in modulating the immune response still remains unclear.

Ayu, *Plecoglossus altivelis*, is an economically important fish species in Asia. In recent years, outbreaks of serious vibriosis caused by *V*. *anguillarum* had been reported in ayu culture [21, 22], resulting in mass mortality. The innate immune system is the first line of fish defense against a broad spectrum of pathogens. Therefore, a deeper understanding of the regulation of the fish’s innate immune response is necessary. In this study, we identified a novel EP4L gene in ayu for the first time. The tissue expression profile of the PaEP4L gene and its association with *V*. *anguillarum* infection was also determined. Moreover, we assessed the function of PaEP4L in ayu MO/MΦ.

## Materials and Methods

### Experimental Animals

About 120 healthy ayu, weighing 40–50 g each, were obtained from a commercial farm in Huangtan Reservoir, Ningbo City, China. The fish were fed daily with pelleted dry food, and acclimatized to laboratory conditions for 2 weeks before the initiation of the experiments. The experiments were approved by the Committee on Animal Care and Use and the Committee on the Ethics of Animal Experiments of Ningbo University.

### Molecular Cloning of PaEP4L cDNA

PaEP4L cDNA sequence was obtained from transcriptome analysis of ayu head kidney-derived MO/MΦ [23]. The authenticity of the PaEP4L cDNA was confirmed by PCR, cloning, and sequencing. The sequence obtained was then compared against other similar known sequences using BLAST search (http://blast.ncbi.nlm.nih.gov/Blast.cgi). Multiple alignments were analyzed using the ClustalW program (http://clustalw.ddbj.nig.ac.jp/) and phylogenetic tree analysis were conducted by the neighbor-joining method using MEGA version 5 [24]. The sequences of fish EP4 used in this study were listed in Table 1.

**Table 1:** EP4 sequences used in this study.

	Species		
Accession no.	Latin name	English name	Protein
BT059530	*Salmo salar*	Atlantic salmon	EP4a
KM502965	*S*. *salar*	Atlantic salmon	EP4b
NM_001039629	*Danio rerio*	zebrafish	EP4a
NM_001128367	*D*. *rerio*	zebrafish	EP4b
NM_001281996	*D*. *rerio*	zebrafish	EP4c
XM_004072297	*Oryzias latipes*	Japanese ricefish	EP4L1
XM_004078943	*O*. *latipes*	Japanese ricefish	EP4L2
NM_001308974	*O*. *latipes*	Japanese ricefish	EP4b
XM_003449642	*Oreochromis niloticus*	Nile tilapia	EP4
XM_007258083	*Astyanax mexicanus*	blind cave fish	EP4L
XM_004549818	*Maylandia zebra*	zebra mbuna	EP4
XM_008325786	*Cynoglossus semilaevis*	tongue sole	EP4
KT873286	*Plecoglossus altivelis*	ayu	EP4L

### Bacterial Infection and Tissue Sampling

*V*. *anguillarum* was grown at 28°C in nutrient broth, and collected in the logarithmic growth phase. Ayu were infected by injecting *V*. *anguillarum* (1.2 × 10^4^ CFU/fish in 100 μl PBS) into the peritoneum for the infected group, while PBS was used in the control group. Heart, liver, spleen, gill, head kidney, and intestine tissue samples were collected at 4, 8, 12, and 24 hours post infection (hpi), after which they were immediately snap-frozen in liquid nitrogen, and preserved at -80°C until subsequent use.

### Prokaryotic Expression

PaEP2L cDNA sequence was obtained from transcriptome analysis of ayu head kidney-derived MO/MΦ. According to the PaEP4L and PaEP2L sequence alignment results, the partial sequence encoding a protein fragment at amino acid position 147–352 of PaEP4L and a protein fragment at amino acid position 163–368 of PaEP2L were amplified using the primer pairs, PaEP4LpF, PaEP4LpR, PaEP2LpF and PaEP2LpR (Table 2). After digestion by BamH I and Xho I, the amplicons were cloned into the pET-30a expression vector, and the constructed plasmids were subsequently transformed into *Escherichia coli* BL21 (DE3). The recombinant proteins with a N-terminal 6 × His-tag (rPaEP4L or rPaEP2L) expressions were induced with IPTG. The recombinant proteins with a N-terminal 6 × His-tag were purified using a nickel-nitrilotriacetic acid (Ni-NTA) column (QIAGEN, Shanghai, China) and resolved by SDS-PAGE.

**Table 2:** Oligonucleotide primers used in this work.

Primer	Gene	Accession number	Nucleotide sequence (5′→3′)	*Amplicon size (base pairs)*
PaEP4LpF	EP4L	KT873286	GGGATCCCTGCCCAGTATGGGTCTTG ^a^	635
PaEP4LpR			GCTCGAGTCAGCAGTCGCGGGACA^a^	
PaEP2LpF	EP2L	JP726299	GGGATCCATGCCGTTCGCTGGATTTG ^b^	632
PaEP2LpR			GCTCGAGTCAGACCATCTGTGTGGG^b^	
PaEP4LF	EP4L	KT873286	ACCCTTTACTGGCCACCTCT	110
PaEP4LR			ATCCGGGAACGTGACATAAA	
PaEP2LF	EP2L	JP726299	CTCCTCCTCCTCTCCGTCAA	203
PaEP2LR			CTGGGCTGAACGAACTCTGT	
PaTNF-αF	TNF-α	JP740414	ACATGGGAGCTGTGTTCCTC	115
PaTNF-αR			GCAAACACACCGAAAAAGGT	
PaIL-1βF	IL-1β	HF543937	TACCGGTTGGTACATCAGCA	104
PaIL-1βR			TGACGGTAAAGTTGGTGCAA	
PaIL-10F	IL-10	JP758157	TGCTGGTGGTGCTGTTTATGTGT	73
PaIL-10R			AAGGAGCAGCAGCGGTCAGAA	
pActin2F	β-actin	AB020884	TCGTGCGTGACATCAAGGAG	231
pActin2R			CGCACTTCATGATGCTGTTG	

^a^ The underlined nucleotides represent the restriction sites for BamH I and Xho I, respectively.

^b^ The underlined nucleotides represent the restriction sites for BamH I and Xho I, respectively.

### Preparation of Anti-PaEP4L IgG

Regarding the interaction of PGE2 with the EP4 receptor, a single threonine residue in the second extracellular loop was identified as being essential [25]. The sequence T^162^WCFIDWRTNDSTHAT coupling to a carrier protein KLH was chosen to be chemically synthesized for the preparation of the rabbit polyclonal antibody (GL Biochem, Shanghai, China). MO/MΦ were incubated with different concentrations of anti-PaEP4L IgG, and then the relative mRNA expression of IL-1β was tested by QPCR. Based on this assay, the optimal concentration of anti-PaEP4L IgG used in neutralization was determined to be 20μg/ml.

### Primary Culture of Ayu Head Kidney-Derived MO/MΦ

Ayu specimens were deeply anaesthetized with ethylene glycol monophenyl ether (0.03% v/v) before the head kidneys were collected aseptically. MO/MΦ from head kidneys were isolated as previously described [26]. Briefly, single cell suspensions of head kidneys were prepared using a 100-μm wire mesh, layered using a Ficoll gradient (Invitrogen, Shanghai, China), and subsequently centrifuged at 400 × g for 25 min. The leukocyte fraction in the Ficoll-medium interface was collected and incubated overnight at 24°C. Non-adherent cells were washed off, and the attached cells were incubated with RPMI 1640 medium containing 5% fetal bovine serum (FBS), 5% ayu serum, 100 U/ml penicillin, and 100 μg/ml streptomycin throughout the experiment. Over 95% of the adherent cells were MO/MΦ according to morphological characteristics observed after Giemsa staining. For cAMP blockade, 30 μM Rp-adenosine 3’,5’-cyclic monophosphorothioate triethylamine salt (Rp-cAMPS) was added to culture medium 2 h before stimulation with PGE2.

### RNA Extraction and First-Strand cDNA Synthesis

Total RNA content was extracted from the tissues using RNAiso reagents (TaKaRa, Dalian, China) according to the manufacturer's instructions. Each extracted RNA sample (5 μg) was incubated with DNase I (TaKaRa) to remove any genomic DNA contamination. Single-stranded cDNA was synthesized from 1 μg of the total RNA using an M-MLV reverse transcriptase (RNase H^-^) (TaKaRa).

### Real-Time Quantitative PCR (QPCR)

QPCR was performed using SYBR premix Ex Taq (Perfect Real Time; TaKaRa) and primer pairs for genes were listed in Table 2. The reaction mix was incubated in a StepOne™ Real-Time PCR System (Applied Biosystems, Foster City, CA, USA) for 500 s at 95°C, followed by 36 amplification cycles of 15 s at 95°C, 15 s at 60°C, and 20 s at 72°C. The experiments were independently replicated four times for each group. Relative mRNA expression of the target gene compared to that of the internal control was calculated using the comparative Ct method (2^-△△Ct^ method) [27]. All data of the QPCR of the relative expression and standard error in this work were shown in S1 Table.

### Anti-CSF1R IgG Staining and Flow Cytometry

MO/MΦ isolated from the head kidney of ayu were washed in FACS buffer (PBS, 0.2% BSA, 0.1% sodium azide), and resuspended to a concentration of 2 × 10^7^/ml. Cells (2 × 10^6^) were incubated with 2 μl anti-CSF1R IgG or 2 μl isotype IgG for 30 min at 4°C. After washing, cells were incubated with the secondary antibody, donkey anti-mouse IgG-PerCP (Jackson ImmunoResearch Laboratories, Westgrove, USA) for 30 min at 4°C, washed, and analyzed by flow cytometry. Data were collected and analyzed on a Gallios Flow Cytometer (Beckman Coulter, Miami, FL, USA).

### Stimulation of MO/MΦ with *V*. *anguillarum* or LPS

Before infection, the medium was changed to antibiotic-free medium and cells were incubated for another 12 h. MO/MΦ were infected with live *V*. *anguillarum* at a multiplicity of infection (MOI) of 2 or LPS at a final concentration of 10 μg/ml. Bacterially-infected or LPS-stimulated cells were harvested at 0, 4, 8, 12, and 24 hpi. Total RNA was extracted from cells using RNAiso reagent (TaKaRa). Simultaneously, cells were also lysed in buffer containing protease inhibitors (20 mM Tris-HCl, 1 mM EDTA, 1% Triton X-100, 1 mM phenylmethanesulfonyl fluoride (PMSF), 10 mg ml^-1^ aprotinin, 10 mg ml^-1^ leupeptin, and 10 mg ml^-1^ pepstatin-A, pH 8.0), and total proteins were prepared as described previously [28].

### Western Blot Analysis

MO/MΦ subjected to bacterial infection were lysed in a buffer (pH 7.4) and the soluble protein concentration was measured using the Bradford method. Western blot analysis and enhanced chemiluminescence (ECL) detection were performed as previously described [26]. Changes in relative band intensity were analyzed by the NIH ImageJ program. Whole original Western blot analysis images of the key immunoblotting experiments were shown in S1 Fig.

### Immunofluorescence Assays

Immunofluorescence assay was performed as previously reported [29]. Briefly, MO/MΦ were fixed in 4% paraformaldehyde for 30 min, then air-dried, followed by soaking in PBS. Cells were blocked with 5% BSA in PBS before incubation with polyclonal rabbit antibody to PaEP4L (1:50 dilution) at 4°C overnight. Cells were visualized with a laser confocal microscope IX81-FV1000 (Olympus, Tokyo, Japan) using R-Phycoerythrin AffiniPure F(ab')_2_ donkey anti-rabbit IgG (1:200, Jackson ImmunoResearch, West Grove, PA, USA) in PBS. DAPI (10 μg/ml, Sigma-Aldrich, Shanghai, China) was used to stain the cell nucleus.

### Stable Expression of PaEP4L and Ligand Binding Assay

PaEP4L cDNA subcloned into the baculovirus transfer vector pBlueBacHis B at the Xho I and Hind III sites. The recombinant transfer vector was sequenced to confirm that the PaEP4L cDNA was correctly inserted. Then the recombinant PaEP4L baculovirus were generated according to the manufacturer's instructions [30]. Briefly, recombinant transfer vectors containing PaEP4L cDNAs were purified by CsCl gradient ultracentrifugation. Sf9 insect cells were seeded in a 6-well plate. When Sf9 cells grew and covered about 50% of the surface, they were ready for viral transfection. Recombinant transfer vectors (3 μg), 1 μg of linear wild-type AcMNPV viral DNA and 20 μl of lipofectin were mixed in 1 ml of culture medium. The medium of Sf9 cells in a well was removed and the 1 ml transfection solution was added to the Sf9 cells in that well. In this way, primary recombinant baculovirus was generated containing PaEP4L cDNAs. MG1 insect cells were used for PaEP4L receptor expression. Fresh MG1 insect cells (5 × 10^6^) were seeded in a 75 cm^2^ tissue-culture flask in 15 ml of complete TNM-FH medium and allowed to grow to 50% confluency. Then recombinant PaEP4L baculovirus, at an MOI of 10, was added to the seeded MG1 cells. The MG1 insect cells were infected for 4 days and were collected from the flask. The membrane fraction of the MG1 cells was prepared as previously described [30]. Briefly, the infected cells were collected and centrifuged for 10 min at 1,000 × g. The cell pellet was resuspended in PBS solution and then sonicated. The sonicated solution was centrifuged at 500 × g for 10 min at 4°C. The supernatant was collected and centrifuged at 100,000 × g for 45 min at 4°C. The membrane pellet was collected and resuspended in PBS.

PaEP4L receptor was assayed for [^3^H]-PGE2 binding with a modified method as described previously [31]. Briefly, membranes were incubated with various amounts of [^3^H]-PGE2 in a final volume of 100 μl of binding buffer (20 mM Tris-HC1, pH 7.0, containing 10 mM MgC1_2_, 1 mM EDTA and 0.1% gelatin) at 24°C for 45 min. The membranes were collected by centrifugation at 20,000 × g for 15 min at 4°C. The membranes were washed with ice-cold binding buffer and again centrifuged. After washing twice with ice-cold binding buffer, the membranes were resuspended in cocktail and counted for radioactivity by liquid scintillation counting. Binding data were analyzed by a computer program (radioligand binding analysis program) to calculate K_d_ from Scatchard plots.

### Measurement of PGE2

PGE2 concentration was determined by using an enzyme-linked immunosorbent assay (Caymen Chemical, Ann Arbor, MI, USA) in accordance with the recommendations of the manufacturer.

### Phagocytosis Assay

*Escherichia coli* DH5α in the logarithmic phase of growth were collected and labeled with fluorescein isothiocyanate (FITC) (Sigma-Aldrich) according to the manufacturer’s protocol and cells were hereafter designated as DH5α-FITC. MO/MΦ grown on cover slips in 6-well plates (2×10^6^ cell/ml) were incubated with 20 μg/ml anti-PaEP4L IgG for 2 h, and then treated with PGE2 for another 16 h. As a control, PBS and rabbit isotype IgG were added. DH5α-FITC were added at an MOI of 20, and cells were further incubated for 30 min before washing extensively with sterile PBS. Trypan blue (0.4%) was used to quench the fluorescence that resulted from particles, which were outside of the cells or sticking to the surface of the cells. Bacterial uptake was then analyzed using a Gallios flow cytometer (Beckman Coulter).

### Respiratory Burst Assay

The respiratory burst of ayu MO/MΦ was measured using a nitro blue tetrazolium (NBT) reduction test as previously described [23]. Briefly, MO/MΦ were incubated with 500 μl PBS containing 0.1% NBT (Sigma-Aldrich) in 35 mm plates for 1.5 h. The reaction was stopped by adding 70% methanol. Then, 130 μl of 2 M KOH and 150 μl of dimethyl sulfoxide (DMSO) were added to the plates to dissolve the formazan. Formazan quantification was performed using an Ultrospec 1100 Pro UV/visible spectrophotometer (Amersham Biosciences, Piscataway, NJ, USA) at 620 nm.

### cAMP Assay

MO/MΦ grown on cover slips in 6-well plates (2×10^6^ cell/ml) were incubated with anti-PaEP4L IgG and 10^−6^ M PGE2. As a control, rabbit isotype IgG and PBS were added. Prior to the assay, cells were washed three times in cold PBS and subsequently lysed in Cell Lysis Buffer provided in the kit. The amount of cAMP was measured using the cAMP parameter assay kit (R&D systems, Minneapolis, MN, USA). The procedure was performed following the manufacturer’s instructions. As this assay is based on competitive binding technique, the value of OD_450_ is inversely proportional to the concentration of cAMP as described previously [20].

### Statistical Analysis

Results are presented as mean ± SEM. All data were analyzed by one-way ANOVA using SPSS version 13.0 (SPSS Inc, Chicago, USA). *P* < 0.05 was considered statistically significant.

## Results

### PaEP4L Gene Analysis

**Fig 1:**
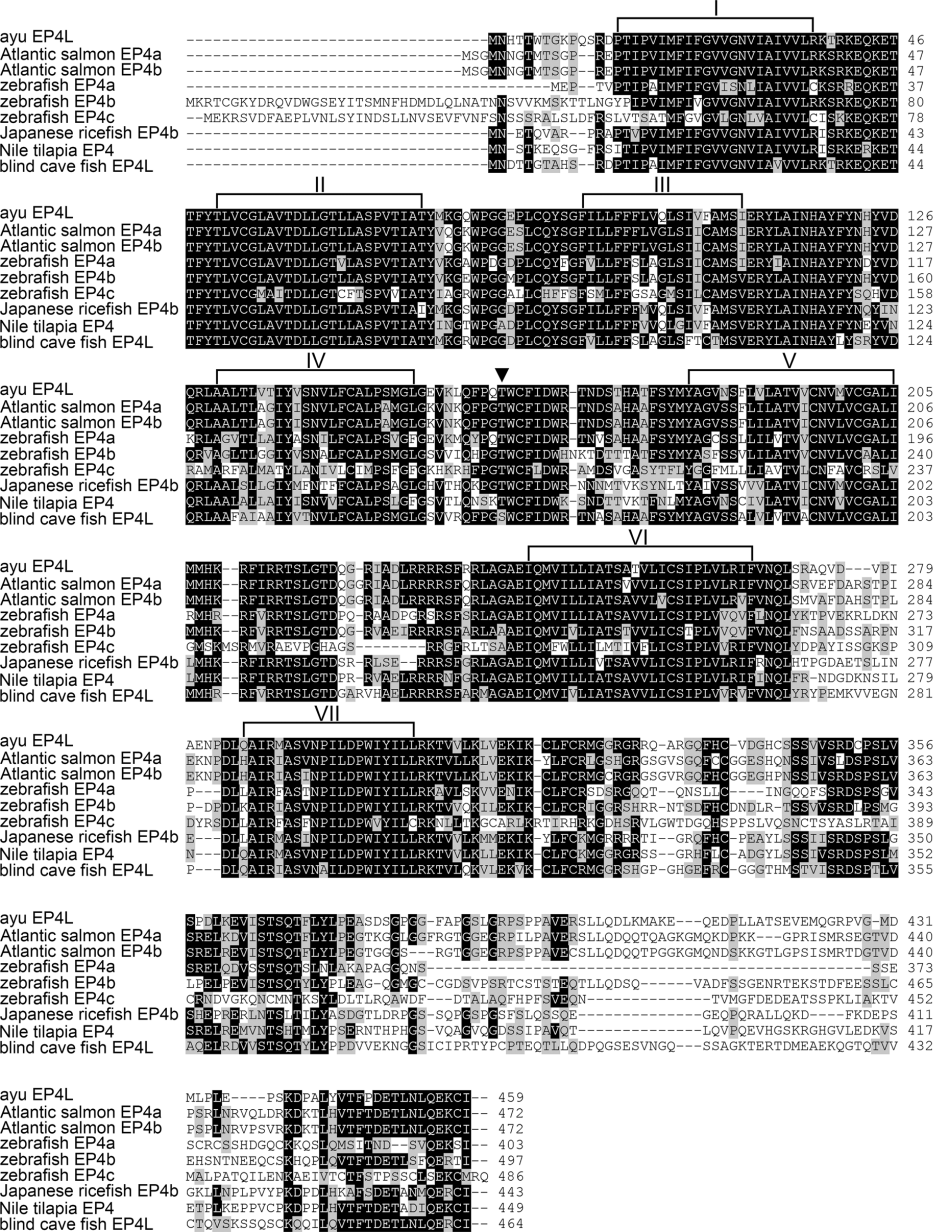
Multiple alignment of the amino acid sequences of PaEP4L and other closely related fish EP4s. Threshold for shading is >60%. Similar residues are marked with gray shading, identical residues are marked with black shading, and alignment gaps are marked as “–”. The PGE2 binding site is marked as ‘‘▼”. The putative transmembrane domains are bracketed and numbered with Roman numerals. Accession numbers of sequences used are listed in Table 1.

**Fig 2:**
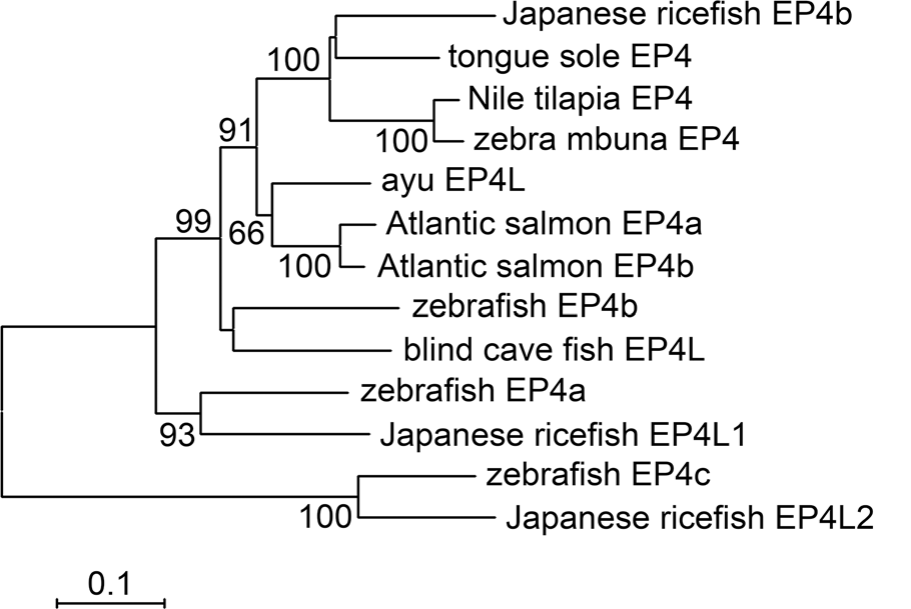
Phylogenetic tree analysis of PaEP4L amino acid sequences and related fish EP4s. The values at the forks indicate the percentage of trees in which this grouping occurred after bootstrapping (1,000 replicates; shown only when >60%). The scale bar shows the number of substitutions per base. Accession numbers of sequences used are listed in Table 1.

PaEP4L cDNA sequence was deposited in GenBank with the accession number: KT873286. It consisted of 2781 nucleotides containing a large open reading frame (ORF) which encoded a 459-aa protein with a molecular weight (MW) of 51.17 kDa and a theoretical isoelectric point (*p*I) of 8.82. A multiple alignment of some fish EP4 amino acid sequences revealed that EP4 were highly conserved at their seven transmembrane domains while N- and C-terminals varied (Fig 1). PaEP4L had a pair of conserved cysteine residues (Cys^86^-Cys^164^) in the second and third extracellular domains (Fig 1), which would form a disulfide bond critical for stabilization of receptor conformation. A single threonine residue, T^162^, in the second extracellular loop of PaEP4L was thought to be essential in the interaction of PGE2 with the EP4 receptor. Sequence comparisons showed that PaEP4L shared the highest amino acid identity (76%) with Atlantic salmon (*Salmo salar*) EP4b. To identify the evolutionary relationship of PaEP4L and other fish, a phylogenetic tree was constructed showed that PaEP4L was most closely related to Atlantic salmon (*Salmo salar*) EP4a and EP4b (Fig 2).

### Alteration of Tissue PaEP4L Transcripts in Response to Infection

**Fig 3:**
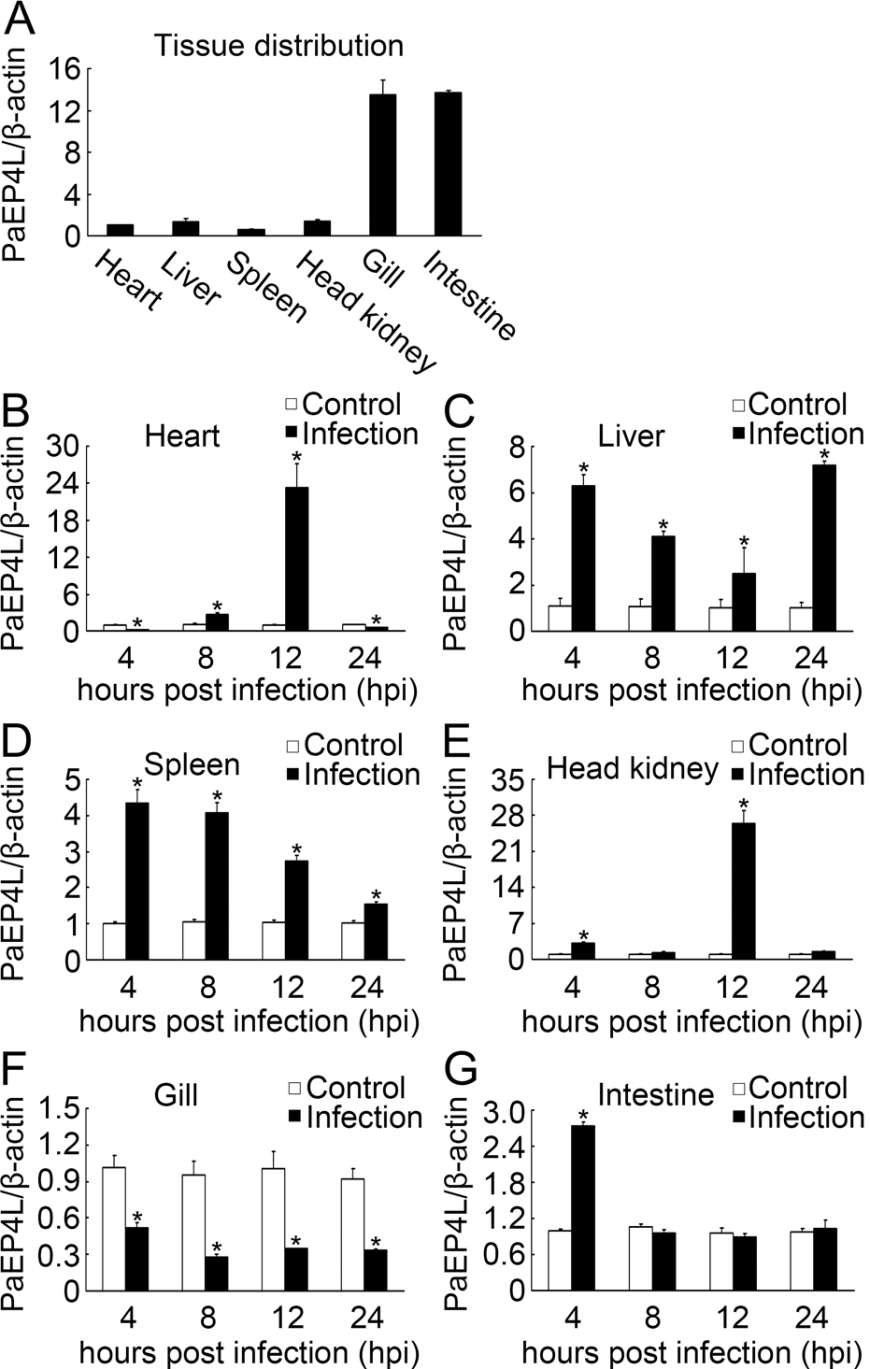
QPCR analysis of PaEP4L mRNA expression in ayu tissues. (A) PaEP4L mRNA expression in healthy ayu tissues. (B-G) PaEP4L mRNA expression in ayu challenged with *V*. *anguillarum*. Tissues were collected at different time points post bacterial infection. PaEP4L mRNA expression was normalized to that of β-actin. Data are expressed as the mean ± SEM of the results from four fish. *p < 0.05.

QPCR was performed to analyze the tissue mRNA expression of PaEP4L in healthy and bacterially infected ayu. In healthy ayu, the PaEP4L transcripts were detected in all six tested tissues with highest expression in intestine and gill (Fig 3A). In ayu infected with *V*. *anguillarum*, PaEP4L mRNA expression was significantly up-regulated in spleen and liver at all sampling time points, in head kidney at 4 and 12 hpi, in heart at 8 and 12 hpi, and in intestine at 4 hpi (Fig 3). By contrast, PaEP4L mRNA expression in gill was down-regulated at all sampling time points (Fig 3F).

### The Specificity of Anti-PaEP4L IgG

**Fig 4:**
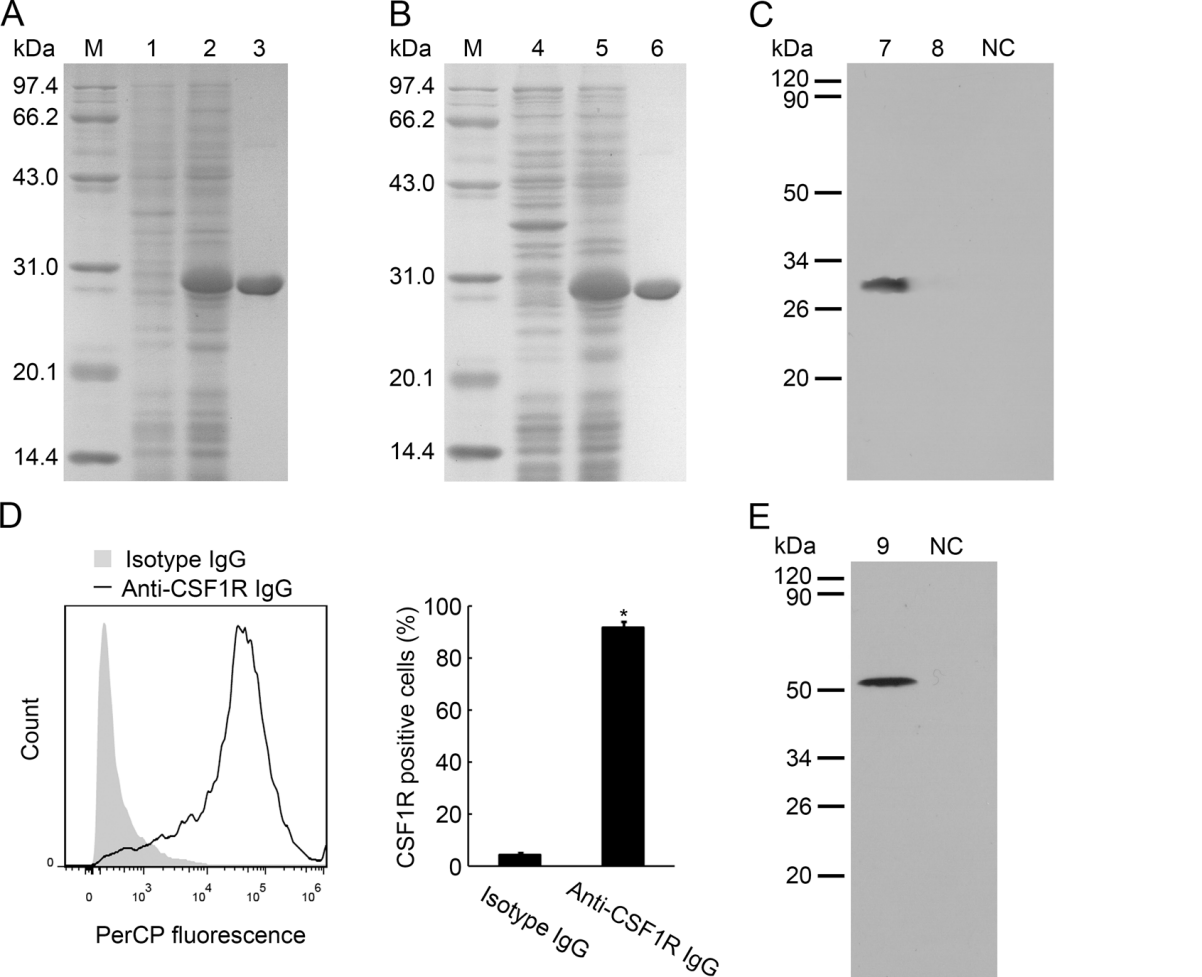
The specificity of the antibody for PaEP4L. (A and B) The SDS-PAGE analysis of prokaryotic expressed PaEP4L and PaEP2L. Lane M: protein marker; 1 and 2: protein from BL21 (DE3) transformed with the pET-30a-PaEP4L plasmid before and after IPTG induction; 3: purified recombinant PaEP4L; 4 and 5: protein from BL21 (DE3) transformed with the pET-30a-PaEP2L plasmid before and after IPTG induction; 6: purified recombinant PaEP2L. (C) Western blot analysis of the specificity of the antibody for PaEP4L. Lane 7: purified recombinant PaEP4L; 8: purified recombinant PaEP2L; NC: negative control. (D) Flow cytometric analysis of the percentage of CSF1R-positive cells in ayu head kidney-derived MO/MΦ following incubation with anti-CSF1R IgG or isotype IgG. Histogram represents the percentage of CSF1R-positive cells in the anti-CSF1R IgG or isotype IgG-treated groups. (E) Western blot analysis of mature PaEP4L protein from MO/MΦ. Lane 9: total proteins of MO/MΦ; NC: negative control.

To investigate the function of PaEP4L in MO/MΦ, we prepared an antibody for PaEP4L and confirmed its specificity. Only PaEP4L and PaEP2L cDNA sequences were obtained from transcriptome analysis of ayu head kidney-derived MO/MΦ [23], suggesting that PaEP4L and PaEP2L were the main receptors of PGE2 in MO/MΦ. So we measured the recombinant proteins (rPaEP4L and rPaEP2L) by anti-PaEP4L IgG to confirm the specificity of anti-PaEP4L IgG. Firstly, we produced the rPaEP4L and rPaEP2L proteins by prokaryotic expression (Fig 4A and 4B). And we found that anti-PaEP4L IgG could recognize the rPaEP4L protein, but not rPaEP2L protein by Western blot analysis (Fig 4C). We further investigated the expression of mature PaEP4L protein from MO/MΦ. Firstly, flow cytometry was used to characterize the head kidney-derived MO/MΦ, and according to the data, 91.58% of MO/MΦ were CSF1R-positive cells (a marker of MO/MΦ in teleosts) (Fig 4D). Western blot analysis in MO/MΦ only recognized one band with molecular weight of 51 kDa (Fig 4E), which was consistent with predicted molecular weight of PaEP4L, but not PaEP2L. These results suggested that anti-PaEP4L IgG was specific to recognize PaEP4L.

### Alteration of PaEP4L and PaEP2L Expression in MO/MΦ upon *V*. *anguillarum* Infection

**Fig 5:**
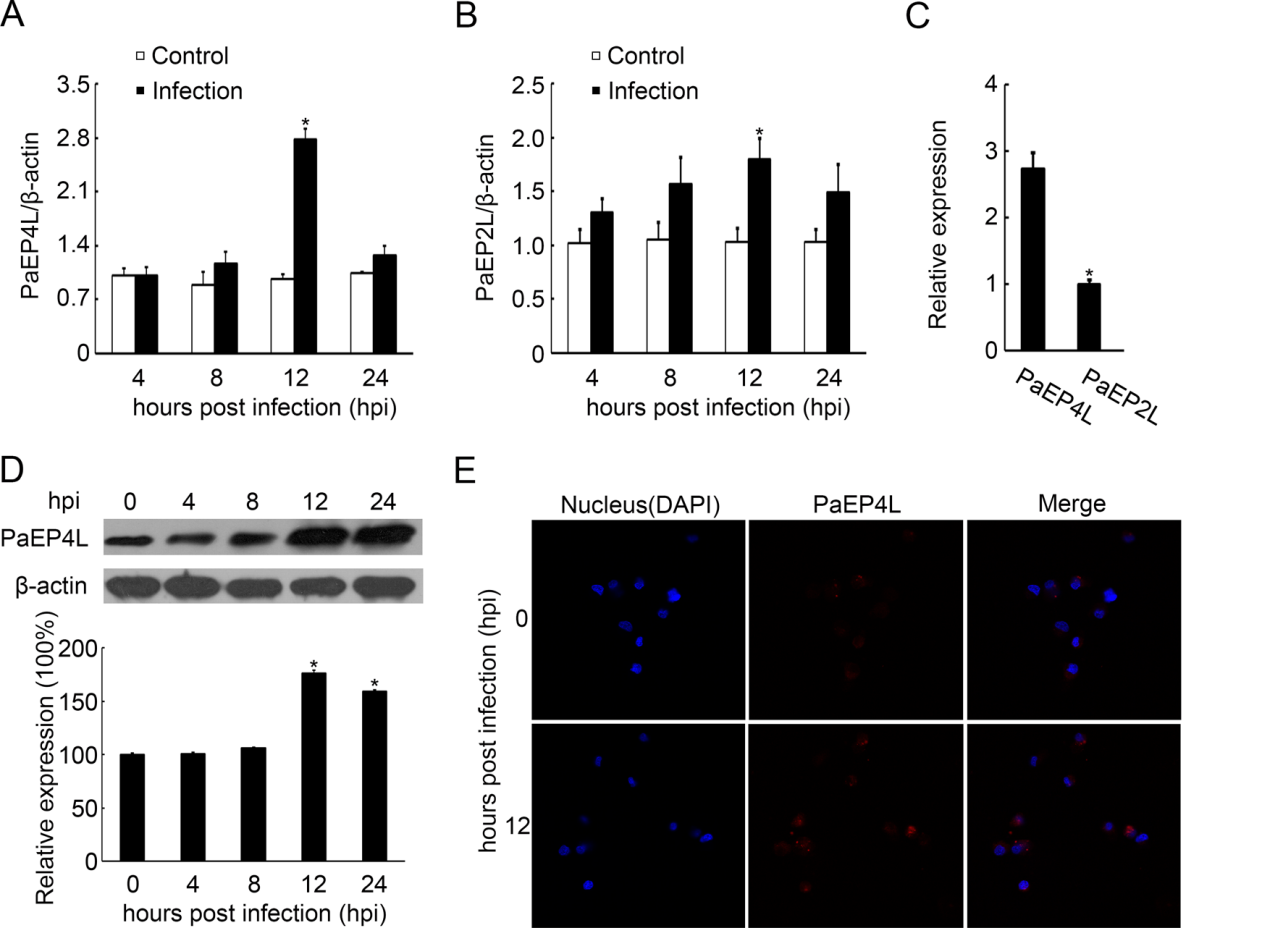
Alteration of PaEP4L and PaEP2L expression in *V*. *anguillarum*-infected ayu head kidney-derived MO/MΦ. Samples were collected at 0, 4, 8, 12 and 24 hpi. (A-C) QPCR was used to analyze PaEP4L and PaEP2L mRNA expression. (D) Western blot analysis was performed to analyze PaEP4L protein expression using an antibody specific to PaEP4L. Histogram displaying the changes in relative band intensity of PaEP4L in samples collected at 0, 4, 8, 12 and 24 hpi. (E) Immunofluorescence assay of PaEP4L in ayu MO/MΦ was performed using a laser confocal microscope. Data are expressed as the mean ± SEM of the results from four fish. *p < 0.05.

We investigated the expression levels of PaEP4L and PaEP2L receptors in healthy and bacterially infected ayu MO/MΦ. Before *V*. *anguillarum* infection, PaEP4L mRNA expression in MO/MΦ was about 2.73 fold compared with PaEP2L (Fig 5C). And *V*. *anguillarum* infection significantly up-regulated (2.84 fold) PaEP4L mRNA expression in MO/MΦ at 12 hpi compared with the control as shown by QPCR (Fig 5A). Although *V*. *anguillarum* infection also up-regulated PaEP2L mRNA expression in MO/MΦ at 12 hpi, the expression was still far less than PaEP4L (Fig 5B). Western blot analysis was employed to detect PaEP4L expression in MO/MΦ. *V*. *anguillarum* infection significantly increased PaEP4L expression at 12 (about 1.76 fold) and 24 hpi (about 1.59 fold), compared with the control (Fig 5D). Immunofluorescence analysis of MO/MΦ also showed PaEP4L expression was increased by *V*. *anguillarum* infection (Fig 5E).

### PaEP4L-Mediated PGE2 Effect on LPS-Stimulated Cytokine Expression in Ayu MO/MΦ

**Fig 6:**
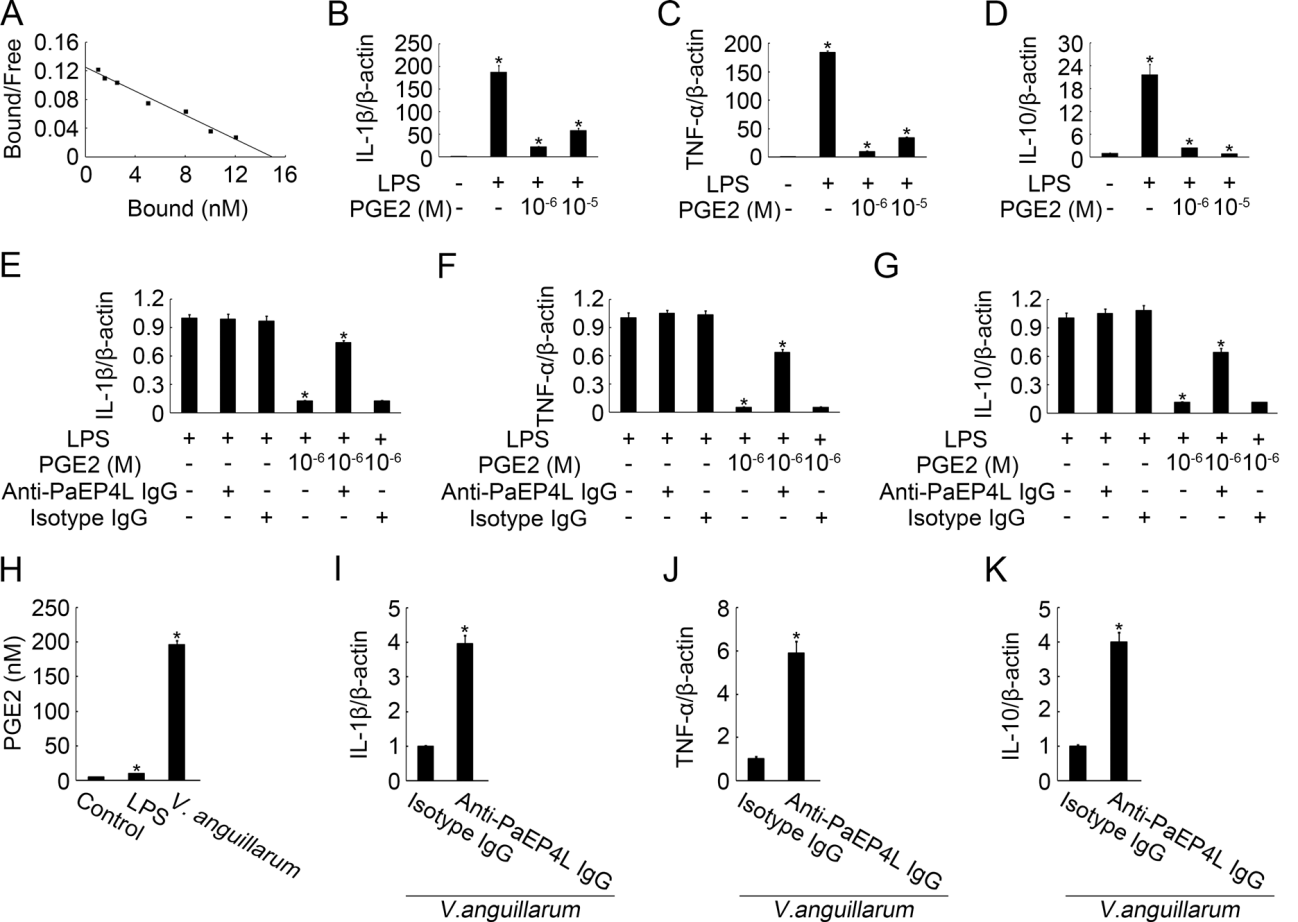
PaEP4L-mediated PGE2 effect on mRNA expression of IL-1β, TNF-α, and IL-10 in ayu MO/MΦ. (A) Scatchard analysis of PGE2 binding of the PaEP4L receptor. The mRNA expression of IL-1β (B), TNF-α (C), and IL-10 (D) in ayu MO/MΦ was determined by QPCR. Following incubation of MO/MΦ with different concentrations of PGE2 for 16 h, cells were stimulated by LPS at a final concentration of 10 μg/ml for another 8 h. The mRNA expression of IL-1β (E), TNF-α (F), and IL-10 (G) was also measured in ayu MO/MΦ under PGE2 and LPS treatment after neutralized by anti-PaEP4L IgG or isotype IgG. (H) Effects of LPS treatment and *V*. *anguillarum* infection on PGE2 production in ayu MO/MΦ. Following incubation of MO/MΦ with anti-PaEP4L IgG or isotype IgG, cells were infected with live *V*. *anguillarum* at an MOI of 2. The mRNA expression of IL-1β (I), TNF-α (J), and IL-10 (K) in ayu MO/MΦ was determined by QPCR. Data are expressed as the mean ± SEM of the results from four fish. *p < 0.05.

Furthermore, we investigated the function of PaEP4L in ayu MO/MΦ. The recombinant PaEP4L receptor had a K_d_ of 120.48 nM for PGE2 (Fig 6A). Therefore we chose 0, 10^−6^, and 10^−5^ M PGE2 to pretreat ayu MO/MΦ. Anti-PaEP4L IgG was added to the cells to block PaEP4L before PGE2 treatment. LPS stimulation resulted in the up-regulation of mRNA expression of IL-1β, TNF-α, and IL-10 in ayu MO/MΦ without PGE2 treatment (Fig 6B–6D), while PGE2 treatment significantly lowered the LPS induction of the three cytokines (Fig 6B–6D). Anti-PaEP4L IgG reversed the PGE2 effect on LPS-stimulated cytokine expression in ayu MO/MΦ (Fig 6E–6G). We further analyzed the effects of LPS treatment and *V*. *anguillarum* infection on PGE2 production in ayu MO/MΦ. PGE2 production in ayu MO/MΦ was increased to 2.06 and 40.79 fold of the control after LPS treatment and *V*. *anguillarum* infection, respectively (Fig 6H), suggesting that PGE2 secretion of *V*. *anguillarum* induction may have autocrine effect. Following incubation of MO/MΦ with anti-PaEP4L or isotype IgG, cells were infected with live *V*. *anguillarum* at an MOI of 2. We found anti-PaEP4L IgG increased the expression of IL-1β, TNF-α, and IL-10 in PGE2 treated MO/MΦ (Fig 6I–6K).

### PaEP4L-Mediated PGE2 Effects on Phagocytosis and Respiratory Burst of Ayu MO/MΦ

**Fig 7:**
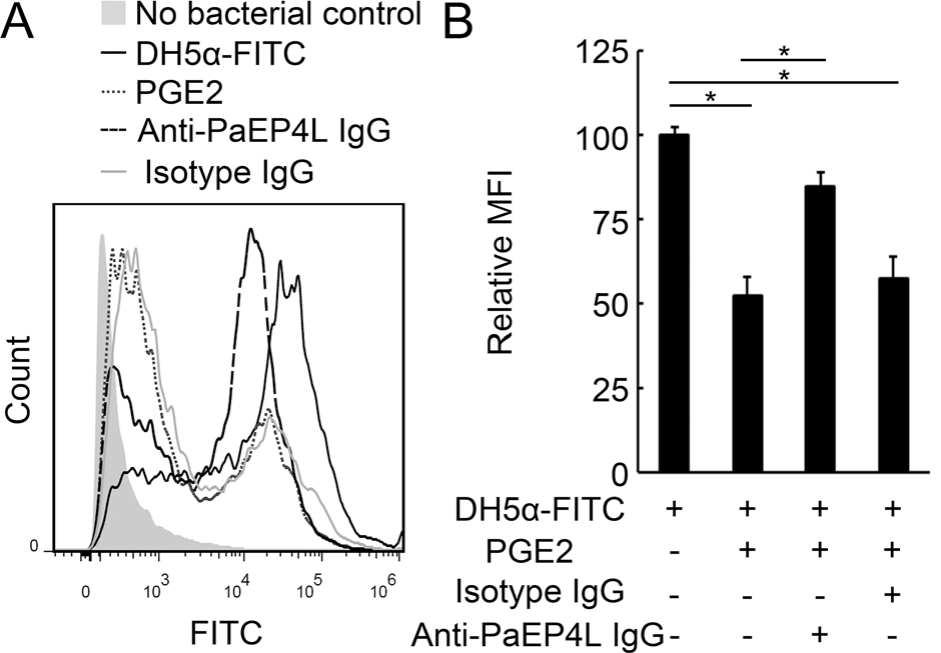
PaEP4L-mediated PGE2 effect on phagocytosis of ayu MO/MΦ. After neutralization by anti-PaEP4L IgG or rabbit isotype IgG for 2 h, ayu MO/MΦ were treated with PGE2 for 16 h. Then DH5α-FITC were added at an MOI of 20, and cells were further incubated for 30 min. (A) Bacterial uptake was analyzed using a Gallios flow cytometer. (B) Relative mean fluorescence intensity (MFI) of PGE2-, anti-PaEP4L IgG- or isotype IgG-treated groups was expressed in fold change relative to the value of PBS-treated control, which was assigned a unit of 100. Data are expressed as the mean ± SEM of the results from four fish. *p < 0.05.

We further analyzed whether PaEP4L mediated PGE2 effects on phagocytosis and respiratory burst of ayu MO/MΦ. MO/MΦ phagocytosis of DH5α-FITC without IgG and PGE2 treatment was set to 100% as the control. MO/MΦ phagocytosis of DH5α-FITC significantly decreased to 52.2% and 57.5% of the control after PGE2 treatment in the IgG-untreated or rabbit isotype IgG-treated groups (Fig 7A and 7B), respectively. Compared with the rabbit isotype IgG-treated group, the phagocytic activity of anti-PaEP4L IgG-treated MO/MΦ increased to 84.9% of the control after PGE2 treatment (Fig 7A and 7B).

**Fig 8:**
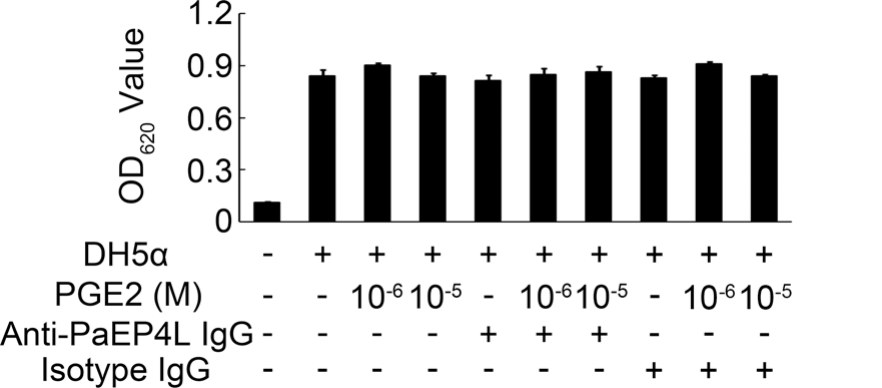
PGE2 and PaEP4L are not involved in respiratory burst of ayu MO/MΦ stimulated by DH5α. Respiratory burst activity was measured using NBT after treatment in ayu MO/MΦ infected with different concentration of PGE2. Data are expressed as the mean ± SEM of the results from four fish. *p < 0.05.

After the neutralization of PaEP4L with anti-PaEP4L IgG, the respiratory burst induced by PGE2 was examined. In DH5α-stimulated groups, the absorbance of 0, 10^−6^, and 10^−5^ M PGE2-treated groups was 0.839 ± 0.037, 0.903 ± 0.012, and 0.839 ± 0.018, respectively. After the neutralization of PaEP4L, the absorbance of 0, 10^−6^, and 10^−5^ M PGE2-treated groups was 0.814 ± 0.030, 0.851 ± 0.033, and 0.865 ± 0.030, respectively. And in isotype IgG groups, the absorbance of 0, 10^−6^, and 10^−5^ M PGE2-treated groups was 0.830 ± 0.014, 0.909 ± 0.012, and 0.840 ± 0.011, respectively (Fig 8).

### PaEP4L-Mediated PGE2 Effects on Ayu MO/MΦ via cAMP Pathway

**Fig 9:**
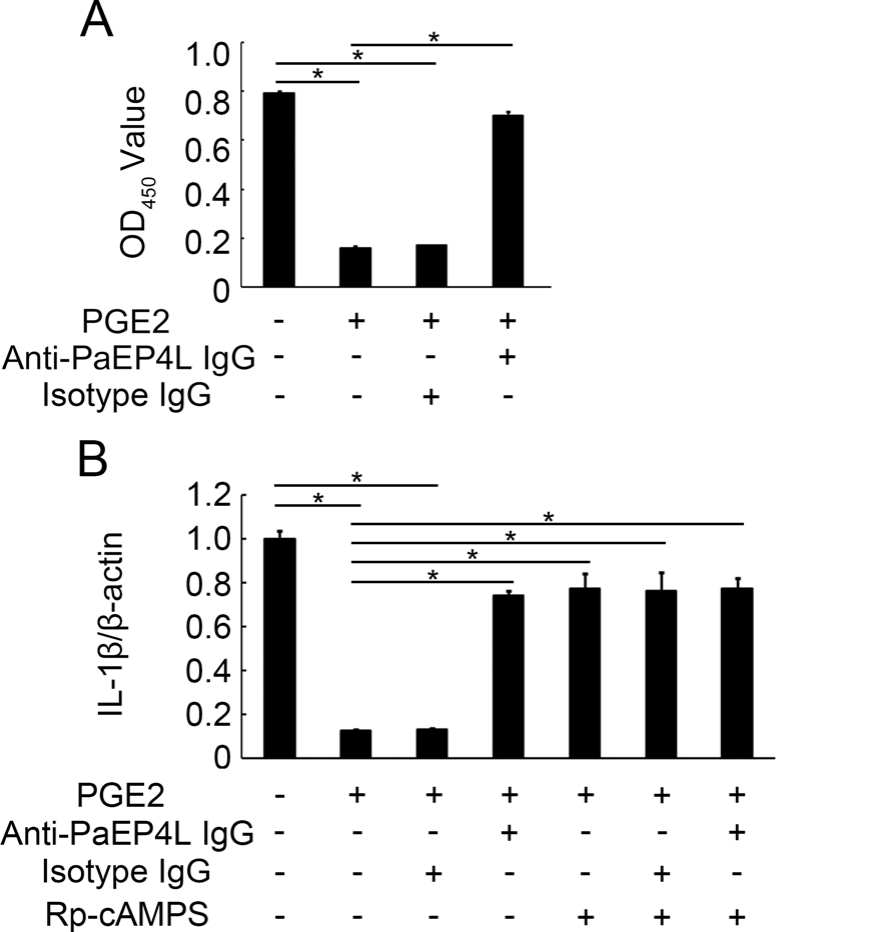
cAMP mediates PGE2/PaEP4L effect on ayu MO/MΦ. (A) cAMP levels were detected after PGE2 treatment. Ayu MO/MΦ were treated with PGE2, anti-PaEP4L IgG or isotype IgG. (B) Anti-PaEP4L IgG and Rp-cAMPS inhibits the PGE2 effect on ayu MO/MΦ. Data are expressed as the mean ± SEM of the results from four fish. *p < 0.05.

To investigate the involvement of cAMP signaling pathway in PGE2 effects on MO/MΦ, the increase of cAMP concentration upon PGE2 and anti-PaEP4L IgG treatment was assayed. In ayu MO/MΦ, cAMP concentration was increased significantly in response to PGE2 treatment (Fig 9A). And anti-PaEP4L IgG reversed the PGE2 effect on cAMP concentration in ayu MO/MΦ (Fig 9A). MO/MΦ were also pretreated with or without anti-PaEP4L IgG, isotype IgG, or Rp-cAMPS, a cAMP competitive analog, prior to stimulation with PGE2. PGE2 treatment significantly decreased the IL-1β expression after LPS induction (Fig 9B). Both Anti-PaEP4L IgG and Rp-cAMPS could reversed the PGE2 effect on LPS-stimulated IL-1β expression in ayu MO/MΦ (Fig 9B), indicating that PaEP4L might signal through cAMP pathway to mediate PGE2 effects on ayu MO/MΦ.

## Discussion

Among the four E-type prostanoid receptors, EP4 is the main receptor responsible for PGE2 function in macrophage [32]. Over the past ten years, numerous studies have revealed that the biological effects of PGE2 can be attributed to EP4 activation [11]. However, the fish PGE2/EP4 signaling pathway remains unclear. In the present study, we obtained the cDNA sequence of an EP4-like protein from ayu. Multiple alignment and phylogenetic tree analysis revealed that PaEP4L belongs to the fish EP4, a member of the family of G protein-coupled receptors. The conservation amongst fish EP4 suggested that they may have similar functions.

Previous studies in Atlantic salmon showed that EP4 mRNA expression was highest in the spleen followed by the head kidney, gill and intestine [19, 20]. In zebrafish, three EP4 genes showed unique expression patterns; and the EP4b transcripts were detected in all tested tissues except for the heart and testis and were highest in the gill [33]. In our study, we found that PaEP4L mRNA was expressed in all tested tissues with high expression in the intestine and gill, which was similar to that reported in other teleosts [19, 20]. In mammals, emerging evidences had shown that chronic inflammation might increase EP4 expression in macrophages [34, 35]. *V*. *anguillarum* infection significantly up-regulated PaEP4L mRNA expression in the liver, spleen, head kidney, and MO/MΦ, which was in accordance with previous reports in mammals [34, 35]. Interestingly, PaEP4L in the gill was down-regulated by bacterial infection, and the reason is not clear.

Inflammatory cytokines, such as IL-1β, IL-10, and TNF-α, are part of the pro- or anti-inflammation immune response [36, 37, 38]. In human primary monocyte-derived macrophages, nucleotide-binding domain, leucine-rich repeat-containing protein (NLR) P3 inflammasome activation is inhibited by PGE2, which is mediated through EP4 [39]. In Atlantic salmon, PGE2 pre-incubation lowered the induction of CXCL-10, CCL-4, IL-8, and IL-1β gene expression in MO/MΦ. However, whether this effect is mediated by EP4 remained unknown [20]. In this study, we found that PGE2 treatment significantly decreased the mRNA expression of IL-1β, IL-10, and TNF-α in ayu MO/MΦ, while anti-PaEP4L IgG neutralization could eliminate PGE2 effect on cytokine expression in MO/MΦ. Some studies showed that the expression of pro- and anti-inflammatory cytokines in mammalian macrophages or fish MO/MΦ were both up-regulated after LPS stimulation [26, 40, 41]. These data revealed that PaEP4L mediated the PGE2 effect on cytokine expression in ayu MO/MΦ. Autocrine PGE2 has been shown to modulate several parameters of mammalian macrophages [42]. However, anti-PaEP4L IgG treatment alone showed no effect on LPS-induced activation. So we further investigated the effects of LPS treatment and *V*. *anguillarum* infection on PGE2 production in ayu MO/MΦ. Although both LPS and *V*. *anguillarum* induced PGE2 up-regulation, PGE2 concentration in LPS treatment group was only about 10 nM and far less than the K_d_ of the recombinant PaEP4L receptor for PGE2, which explain that anti-PaEP4L IgG treatment alone showed no effect on LPS-induced activation in ayu MO/MΦ. Furthermore, anti-PaEP4L IgG treatment led to the up-regulation of inflammatory cytokine in *V*. *anguillarum*-infected MO/MΦ. Hence, PGE2 has an autocrine role in *V*. *anguillarum*-infected MO/MΦ.

The phagocytosis of fish MO/MΦ plays an important role in inflammatory response to bacterial infection [43, 44]. A previous study in human macrophages showed that EP4 mediates the PGE2-induced intracellular cAMP increase and phagocytosis inhibition [45]. The rapid release of reactive oxygen species (ROS) from macrophages or other types of immune cells is known as a respiratory burst. The respiratory burst is partially suppressed by PGE2 treatment in human neutrophils [46]. In our study, the phagocytosis of anti-PaEP4L IgG-treated MO/MΦ was significantly higher than that of isotype IgG-treated MO/MΦ, suggesting that PaEP4L mediated the PGE2 effect on the phagocytosis of ayu MO/MΦ. PGE2 and PaEP4L had no significant effect on the respiratory burst of ayu MO/MΦ. In mammals, PGE2 inhibits the respiratory burst of immune cells and suppresses the release of TNF-α [46–48]. The inflammatory cytokines, such as TNF-α, play important roles in the functional induction of the respiratory burst [49]. However, some teleost fish TNF-α does not directly activate phagocytes [49], suggesting that the mechanism of respiratory burst in fish is different with that in mammals. Our results also showed that PGE2 and PaEP4L affected the expression of TNF-α in MO/MΦ. Hence, up-regulation of the respiratory burst in MO/MΦ by *E*.*coli* in ayu may result from an unknown pathway that is not affected by PGE2 and PaEP4L.

Most PGE2 effects on immune cells have been attributed to rises in cAMP level, as a result of binding of PGE2 to EP4 receptors [50–52]. A previous study in fish-derived cell lines (CHSE-214 and RTG-2 cells, both from salmonid species) both showed strong cAMP increase in response to PGE2 treatment. However, no significant increase was detected in Atlantic salmon TO cells, a macrophage-like cell line [20]. In our study, cAMP concentration was increased significantly in response to PGE2 treatment. Hence, the downstreams of PGE2/EP4 are different in various cell types. Furthermore, the up-regulation of IL-1β expression in PGE2 treated MO/MΦ were found after anti-PaEP4L IgG or cAMP inhibition treatment. These data suggest that cAMP is the downstream of PGE2/PaEP4L to mediate the anti-inflammatory effects of PGE2.

In summary, we identified a novel EP4 like gene in ayu. PaEP4L mediated the PGE2 effects on the regulation of ayu MO/MΦ function in response to bacterial infection. This study enriches our current knowledge of the fish PGE2/EP4 pathway. Further studies are needed to determine the detailed mechanism underlying fish EP4 function in the regulation of the immune response.

## Supporting Information

S1 Fig Original Western blot analysis images of the key immunoblotting experiments. (TIF)

S1 Table Data of the QPCR of the relative expression and standard error in this work. (DOC)
