# Supplementary figures and images for: Molecular Characterization of E-Type Prostanoid Receptor 4 (EP4) from Ayu (Plecoglossus altivelis) and Its Functional Analysis in the Monocytes/Macrophages
Source: PLoS One. 2016 Jan 25;11(1):e0147884. doi: 10.1371/journal.pone.0147884 (PMC4726814; doi:10.1371/journal.pone.0147884)

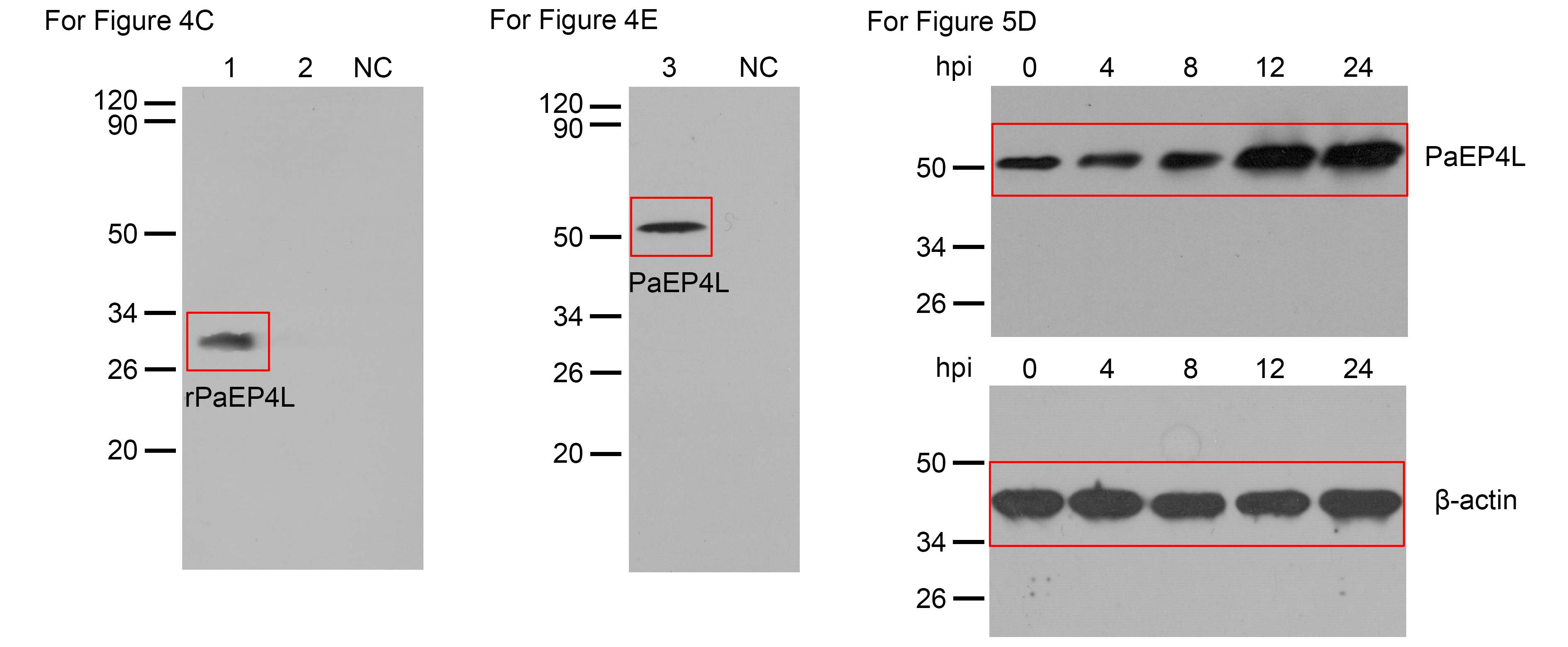

Supplement: S1 Fig — (TIF) [file pone.0147884.s001.tif]
