# Supplementary material for: Molecular Characterization of E-Type Prostanoid Receptor 4 (EP4) from Ayu (Plecoglossus altivelis) and Its Functional Analysis in the Monocytes/Macrophages
Source: PLoS One. 2016 Jan 25;11(1):e0147884. doi: 10.1371/journal.pone.0147884 (PMC4726814; doi:10.1371/journal.pone.0147884)
Supplement: S1 Table — (DOC) [file pone.0147884.s002.doc]

For Figure 3A

|  | PaEP4L | |
| --- | --- | --- |
| Test tissues | Relative expression | Standard error |
| Heart | 1.00 | ±0.038 |
| Liver | 1.31 | ±0.371 |
| Spleen | 0.59 | ±0.029 |
| Head kidney | 1.41 | ±0.115 |
| Gill | 13.53 | ±1.400 |
| Intestine | 13.69 | ±0.212 |

For Figure 3B

| Heart | PaEP4L (Control) | | PaEP4L (Infection) | |
| --- | --- | --- | --- | --- |
| Hours post infectin | Relative expression | Standard error | Relative expression | Standard error |
| 4 | 1.00 | ±0.038 | 0.24 | ±0.008 |
| 8 | 1.08 | ±0.079 | 2.65 | ±0.224 |
| 12 | 1.00 | ±0.031 | 23.32 | ±3.926 |
| 24 | 1.04 | ±0.068 | 0.63 | ±0.020 |

For Figure 3C

| Liver | PaEP4L (Control) | | PaEP4L (Infection) | |
| --- | --- | --- | --- | --- |
| Hours post infectin | Relative expression | Standard error | Relative expression | Standard error |
| 4 | 1.11 | ±0.314 | 6.28 | ±0.488 |
| 8 | 1.08 | ±0.321 | 4.10 | ±0.229 |
| 12 | 1.02 | ±0.337 | 2.51 | ±1.106 |
| 24 | 1.01 | ±0.229 | 7.17 | ±0.185 |

For Figure 3D

| Spleen | PaEP4L (Control) | | PaEP4L (Infection) | |
| --- | --- | --- | --- | --- |
| Hours post infectin | Relative expression | Standard error | Relative expression | Standard error |
| 4 | 1.01 | ±0.050 | 4.35 | ±0.382 |
| 8 | 1.07 | ±0.062 | 4.08 | ±0.285 |
| 12 | 1.05 | ±0.048 | 2.74 | ±0.155 |
| 24 | 1.03 | ±0.060 | 1.54 | ±0.059 |

For Figure 3E

| Head kidney | PaEP4L (Control) | | PaEP4L (Infection) | |
| --- | --- | --- | --- | --- |
| Hours post infectin | Relative expression | Standard error | Relative expression | Standard error |
| 4 | 1.01 | ±0.082 | 3.19 | ±0.103 |
| 8 | 1.01 | ±0.079 | 1.27 | ±0.216 |
| 12 | 1.00 | ±0.076 | 26.35 | ±2.496 |
| 24 | 0.99 | ±0.082 | 1.53 | ±0.187 |

For Figure 3F

| Gill | PaEP4L (Control) | | PaEP4L (Infection) | |
| --- | --- | --- | --- | --- |
| Hours post infectin | Relative expression | Standard error | Relative expression | Standard error |
| 4 | 1.01 | ±0.105 | 0.52 | ±0.044 |
| 8 | 0.95 | ±0.112 | 0.28 | ±0.021 |
| 12 | 1.01 | ±0.139 | 0.35 | ±0.002 |
| 24 | 0.92 | ±0.088 | 0.34 | ±0.003 |

For Figure 3G

| Intestine | PaEP4L (Control) | | PaEP4L (Infection) | |
| --- | --- | --- | --- | --- |
| Hours post infectin | Relative expression | Standard error | Relative expression | Standard error |
| 4 | 1.00 | ±0.016 | 2.74 | ±0.063 |
| 8 | 1.05 | ±0.051 | 0.95 | ±0.051 |
| 12 | 0.96 | ±0.089 | 0.89 | ±0.061 |
| 24 | 0.98 | ±0.049 | 1.03 | ±0.137 |

For Figure 5A

| monocytes/macrophages | PaEP4L (Control) | | PaEP4L (Infection) | |
| --- | --- | --- | --- | --- |
| Hours post infectin | Relative expression | Standard error | Relative expression | Standard error |
| 4 | 1.01 | ±0.090 | 1.02 | ±0.100 |
| 8 | 0.89 | ±0.163 | 1.17 | ±0.145 |
| 12 | 0.97 | ±0.049 | 2.77 | ±0.144 |
| 24 | 1.04 | ±0.016 | 1.27 | ±0.124 |

For Figure 5B

| monocytes/macrophages | PaEP2L (Control) | | PaEP2L (Infection) | |
| --- | --- | --- | --- | --- |
| Hours post infectin | Relative expression | Standard error | Relative expression | Standard error |
| 4 | 1.02 | ±0.123 | 1.30 | ±0.134 |
| 8 | 1.06 | ±0.159 | 1.56 | ±0.254 |
| 12 | 1.03 | ±0.135 | 1.79 | ±0.207 |
| 24 | 1.03 | ±0.125 | 1.49 | ±0.256 |

For Figure 5C

| monocytes/macrophages | PaEP2L | | PaEP4L | |
| --- | --- | --- | --- | --- |
| Hours post infectin | Relative expression | Standard error | Relative expression | Standard error |
| 0 | 1.01 | ±0.060 | 2.73 | ±0.244 |

For Figure 6B

| monocytes/macrophages | IL-1β | |
| --- | --- | --- |
| Groups | Relative expression | Standard error |
| Control | 1.00 | ±0.100 |
| LPS | 172.52 | ±13.46 |
| LPS+10^-6^ M PGE2 | 22.42 | ±0.678 |
| LPS+10^-5^ M PGE2 | 56.30 | ±3.81 |

For Figure 6C

| monocytes/macrophages | TNF-α | |
| --- | --- | --- |
| Groups | Relative expression | Standard error |
| Control | 1.02 | ±0.112 |
| LPS | 183.93 | ±2.82 |
| LPS+10^-6^ M PGE2 | 9.80 | ±0.805 |
| LPS+10^-5^ M PGE2 | 33.40 | ±1.86 |

For Figure 6D

| monocytes/macrophages | IL-10 | |
| --- | --- | --- |
| Groups | Relative expression | Standard error |
| Control | 1.00 | ±0.038 |
| LPS | 20.55 | ±1.497 |
| LPS+10^-6^ M PGE2 | 2.39 | ±0.159 |
| LPS+10^-5^ M PGE2 | 0.80 | ±0.063 |

For Figure 6E

| monocytes/macrophages | IL-1β | |
| --- | --- | --- |
| Groups | Relative expression | Standard error |
| LPS | 1.00 | ±0.037 |
| LPS+Anti-PaEP4L IgG | 0.99 | ±0.052 |
| LPS+Isotype IgG | 0.97 | ±0.053 |
| LPS+10^-6^ M PGE2 | 0.13 | ±0.002 |
| LPS+10^-6^ M PGE2+ Anti-PaEP4L IgG | 0.74 | ±0.021 |
| LPS+10^-6^ M PGE2+ Isotype IgG | 0.13 | ±0.002 |

For Figure 6F

| monocytes/macrophages | TNF-α | |
| --- | --- | --- |
| Groups | Relative expression | Standard error |
| LPS | 1.01 | ±0.054 |
| LPS+Anti-PaEP4L IgG | 1.05 | ±0.029 |
| LPS+Isotype IgG | 1.03 | ±0.046 |
| LPS+10^-6^ M PGE2 | 0.05 | ±0.006 |
| LPS+10^-6^ M PGE2+ Anti-PaEP4L IgG | 0.64 | ±0.033 |
| LPS+10^-6^ M PGE2+ Isotype IgG | 0.05 | ±0.005 |

For Figure 6G

| monocytes/macrophages | IL-10 | |
| --- | --- | --- |
| Groups | Relative expression | Standard error |
| LPS | 1.00 | ±0.052 |
| LPS+Anti-PaEP4L IgG | 1.05 | ±0.049 |
| LPS+Isotype IgG | 1.08 | ±0.053 |
| LPS+10^-6^ M PGE2 | 0.11 | ±0.006 |
| LPS+10^-6^ M PGE2+ Anti-PaEP4L IgG | 0.64 | ±0.040 |
| LPS+10^-6^ M PGE2+ Isotype IgG | 0.11 | ±0.004 |

For Figure 6I

| monocytes/macrophages | IL-1β (Infection) | |
| --- | --- | --- |
| Groups | Relative expression | Standard error |
| PGE2+Isotype IgG | 1.00 | ±0.019 |
| PGE2+ Anti-PaEP4L IgG | 3.97 | ±0.241 |

For Figure 6J

| monocytes/macrophages | TNF-α (Infection) | |
| --- | --- | --- |
| Groups | Relative expression | Standard error |
| PGE2+Isotype IgG | 1.02 | ±0.102 |
| PGE2+ Anti-PaEP4L IgG | 5.91 | ±0.521 |

For Figure 6K

| monocytes/macrophages | IL-10 (Infection) | |
| --- | --- | --- |
| Groups | Relative expression | Standard error |
| PGE2+Isotype IgG | 1.00 | ±0.039 |
| PGE2+ Anti-PaEP4L IgG | 4.00 | ±0.279 |

For Figure 9B

| monocytes/macrophages | IL-1β | |
| --- | --- | --- |
| Groups | Relative expression | Standard error |
| LPS | 1.00 | ±0.037 |
| LPS+10^-6^ M PGE2 | 0.13 | ±0.002 |
| LPS+10^-6^ M PGE2+ Isotype IgG | 0.13 | ±0.003 |
| LPS+10^-6^ M PGE2+ Anti-PaEP4L IgG | 0.74 | ±0.021 |
| LPS+10^-6^ M PGE2+Rp-cAMPS | 0.78 | ±0.069 |
| LPS+10^-6^ M PGE2+Isotype IgG+ Rp-cAMPS | 0.77 | ±0.084 |
| LPS+10^-6^ M PGE2+ Anti-PaEP4L IgG + Rp-cAMPS | 0.77 | ±0.048 |
